# Sensory Experiences and Children With Severe Disabilities: Impacts on Learning

**DOI:** 10.3389/fpsyg.2022.875085

**Published:** 2022-04-29

**Authors:** Susan Agostine, Karen Erickson, Charna D’Ardenne

**Affiliations:** Center for Literacy and Disability Studies, University of North Carolina at Chapel Hill, Chapel Hill, NC, United States

**Keywords:** severe disabilties, grounded theory, post-critical design, sensory experiences, special education

## Abstract

The human sensory system is continuously engaged in experiencing and interpreting every interaction with other living beings, objects, and the environment. The purpose of this article is to describe the impact limited opportunities for rich sensory experiences have on students with severe disabilities in two middle school classrooms situated in a public separate school in the southeastern USA. The study employed a postcritical ethnographic approach and grounded theory thematic analysis of fieldnotes gathered over a two-year period. Three major themes supported by the data are presented and discussed in depth. They are: (a) students are afforded limited sensory rich experiences, (b) everyday routines make students passive recipients to school, and (c) instructional approaches result in little interaction with extended periods of waiting. The implications of the findings for improved sensory experiences and possible future directions are described.

## Introduction

“*Sensation is the common language by which we share the experience of being human; it provides a common ground for understanding*” (Dunn, 2001, p. 608).

Students with severe disabilities present with a variety of physical, sensory, cognitive, and communication needs that impact the ways they interact with and experience the world (Erickson and Geist, 2016). Though there are differences in the ways individual students with a range of abilities seek or avoid sensation (Dunn, 2001), and there are differences in the ways that various contexts place demands on sensation (Dunn, 2007), little is documented regarding the ways that students with severe disabilities experience and interpret their interactions with other humans, objects, and the environment. This study explored the sensory experiences of a group of students with severe disabilities in two middle school special education classrooms situated in a public separate school in the southeastern United States.

### About Children With Severe Disabilities in United States Public Schools

In this manuscript, we discuss *children with severe disabilities*. By this we mean the group of children in United States public schools who receive special education services under the eligibility category of Multiple Disabilities as defined by the Individuals with Disabilities Education Act (2004). The group of children with severe disabilities also includes some children who receive services under the categories of autism, intellectual disability, or some other category (Erickson and Geist, 2016) and have a concurrent severe intellectual disability (American Association of Intellectual and Developmental Disabilities, 2017). In the United States, most children with severe disabilities are educated in special education classrooms or separate schools that exclusively serve children with disabilities (Morningstar et al., 2017; Burnes and Clark, 2021). Although they have diverse cognitive, motor, and sensory profiles (Towles-Reeves et al., 2012; Erickson and Geist, 2016), children with severe disabilities all consistently require: (a) instruction that is extensive, intensive, and individualized, (b) materials that are substantially adapted and modified, and (c) methods of accessing information that are individualized to help them acquire, maintain, generalize, and transfer skills across settings (Dynamic Learning Maps Consortium, 2016; Taub et al., 2017). Children with severe disabilities exhibit a broad range of expressive communication skills. Depending on the source (Towles-Reeves et al., 2012; Erickson and Geist, 2016; Burnes and Clark, 2021), approximately 7–10% percent communicate at a pre-symbolic level (e.g., gestures, vocalizations, facial expressions, and body language for highly contextualized purposes), 18%–31% at an emerging symbolic level (e.g., use of single words, signs, or graphic symbols for a restricted range of purposes), and 61%–69% at a symbolic level (e.g., combining two or more words, signs, or graphic symbols). All of the 25%–41% who communicate at pre-symbolic or emerging levels and 8%–10% who use augmentative and alternative communication to communicate at a symbolic level are said to have complex communication needs (CCN; Erickson and Geist, 2016).

### The Challenge of Sensory Experience for Children With Severe Disabilities

There is a profound lack of literature regarding the sensory experiences of children with severe disabilities. A significant portion of the sensory literature addresses children with autism spectrum disorder or children without disabilities (Ayres and Tickle, 1980; Watling and Dietz, 2007; Engel-Yeger and Dunn, 2011; Pfeiffer et al., 2011; Lang et al., 2012; Mills et al., 2016; Roberts et al., 2018). However, the existing literature serves to inform understandings of the challenge of sensory experiences for children with severe disabilities.

Beginning in infancy, severe disabilities can profoundly delay or preclude the achievement of typical developmental milestones. The altering and delaying of this development affect a child’s world view and sensory development. According to Pexman (2019), children’s physical development is directly linked to how they interact with objects and the ways that conceptual understanding emerges from sensorimotor experience. As gross motor skills improve, infants have more opportunities to manipulate objects in space. Thus, they have new visual and tactile experiences that give them information and feedback about the world. Limited improvements in gross motor skills prevent children with severe disabilities from seeking and manipulating objects in space (Nilsson and Nyberg, 2003). These limitations have cascading effects on visual and tactile experiences and subsequent sensory development (Lima et al., 2013).

One adverse consequence of severe disabilities is limited opportunity to engage physically in play. As described by Parham and Fazio (2008), play facilitates learning and is one of the main occupations of early childhood. They define play as “any spontaneous or organized activity that provides enjoyment, entertainment, amusement or diversion” (p. 448). Play is intrinsically motivated, generally focused on process more than outcome, and integrally related to sensory processing skills among children without disabilities (Roberts et al., 2018). “Through play, children learn sensorimotor rules, rules of objects and of people, and rules of thinking” (Parham and Fazio, 2008, p. 12). Once children understand these initial rules, they build upon them to understand the more complex and interweaving rules of their culture. The importance of play cannot be overstated; however, there is a lack of evidence regarding play and its role in sensory processing and sensorimotor development in children with severe disabilities. What is known is that play has different forms for children with severe disabilities given the limits in their ability to physical interact with objects (Wenger et al., 2021), move their own bodies (Graham et al., 2019), and talk or otherwise interact with others (Clarke and Wilkinson, 2009). This in turn is likely to impact the sensory development that is promoted by typical play.

Whether in play or other interactions, children independently learn how the world works through *sense-making* and, when they have the benefit of interactions with other humans, *participatory sense-making* (Di Paolo and De Jaegher, 2012). As a general concept, sense-making is the creation of meaning through interactions with the world. Individuals use their past experiences to embody current experiences and make meaning. This gives the individual perspective that then shapes how they see the world. Sense-making is a constant and never-ending process that allows people to participate actively in the world. Participatory sense making goes beyond sense-making by emphasizing the ways that two or more people come together to make meaning from the world in a different way than they would do alone. Humans are driven to coordinate with each other in their sense-making in a fluid and dynamic way, and the coordination of two or more physical bodies helps to embody a different perspective on the world. As two or more people work together successfully coordinating their sense-making, they become more in tune with each other “swaying into and out of states that are close to stable, but not quite” (De Jaegher and Di Paolo, 2007, p. 491). Both sense-making and participatory sense-making are important tools in growth and development. When a child has severe disabilities, opportunities for sense-making may be diminished, which leaves them more dependent on participatory sense-making than other children. Thus, the opportunities adults provide for participatory sense-making are uniquely important for children with severe disabilities. While this has been reported anecdotally, no research could be located linking physical development, sensorimotor experience, and conceptual development in children with severe disabilities.

### The Role of Sensory Experience in Learning and Development

How people process sensory information and what happens when they have impairments with sensory processing has been a topic of discussion since the early 1960’s. Ayres (1973) first coined the term *sensory integration* to describe a theory created to “explain the relationship between deficits in interpreting sensation from the body and the environment and difficulties with academic or motor learning” (Bundy et al., 2002, p. 3). Later, Ayres and Robbins (1979) defined sensory integration as:

the organization of sensory input for use. The ‘use’ may be perception of the body or the world, or an adaptive response, or a learning process, or the development of some neural function. Through sensory integration, the many parts of the nervous system work together so that a person can interact with the environment effectively and experience appropriate satisfaction (p. 184).

Since Ayres’ early work, many occupational therapists have expanded upon and critiqued the theory of sensory integration (Wilbarger and Wilbarger, 1991; Dunn, 1997; Bundy et al., 2002; Dunn, 2007). The model of sensory processing by Dunn (1997), which depicts a relationship between the nervous system’s thresholds and self-regulation strategies, informs the work reported in this manuscript. In this model, Dunn (2007) defined a neurological threshold as the point at which a nerve cell or a system has enough input to activate. Each individual’s sensory systems can have different neurological thresholds. For example, an individual might have a high neurological threshold for auditory input (e.g., they can listen to very loud music) but have a very low neurological threshold for tactile input (e.g., light touch is experienced as noxious). Neurological thresholds are related to self-regulation, which is described as the central nervous system’s ability to modulate and respond to the sensations received (Dunn, 1997, 2001). Self-regulation strategies are described on a continuum from passive to active. Passive strategies allow the sensory input to happen without trying to change the environment or the individual. Active strategies involve efforts to control the sensory input to support better self-regulation. These self-regulation strategies directly interact with an individual’s neurological thresholds to create four basic sensory patterns.

As described by Dunn (2007), these patterns are: sensory seeking, sensation avoiding, sensory sensitivity, and low registration. Sensory seeking indicates a high neurological threshold and active self-regulation strategies, and it often results in children who engage in high levels of activity (e.g., never staying in their seats), have a limited of awareness of space (e.g., crashing into things), and high distractibility, which causes them to lose track of daily tasks. Sensory avoiding indicates a low neurological threshold and active self-regulation strategies. Sensory avoiding often results in children hiding and covering their ears when things get loud, crowded, and overwhelming. Sensory sensitivity indicates a low neurological threshold and passive self-regulation. Children with sensory sensitivity get overwhelmed like children who are sensory avoiding, but they have limited active self-regulation, which keeps them from hiding, covering their ears, or otherwise seeking to limit the sensory input, thereby a frequent response to sensory overload can be irritability, being short tempered, or demanding. The final pattern, low registration, indicates high neurological thresholds and passive self-regulation. Children with low registration often sit quietly, apparently unaffected by sensory input, often missing instructions, and doing nothing about it.

Children with low registration usually need adults to work hard to recruit their attention (e.g., calling their name multiple times or touching them). Children with low registration may seem oblivious to their environment and often appear unresponsive in situations that would typically elicit responses from children. Finally, children with low registration rarely yell or call out and are not thought of as having behavior issues that requires a lot of teacher attention. While Dunn (1997) originally described these patterns using data from children without disabilities, the patterns have since been utilized to understand the sensory processing patterns of at-risk children and children diagnosed with disabilities such as autism, ADHD, and Fragile X syndrome (Dunn, 2007).

Severe disabilities have a ripple effect on the development of sensory processing and the ability to enact active patterns in response to sensory input. Limited gross motor movement restricts opportunity to explore the environment, which leads to limited sensorimotor experience needed to make sense of the objects. This then delays fine motor skill development and restricts play, which further restricts sensory development. These motor impairments further restrict access to the active strategies required by some of the sensory seeking and sensation avoiding patterns by Dunn (2007). Participatory sense making is one means of supporting purposeful sensory experience and patterns of sensory processing, but it is vital to also support children with severe disabilities in independent play and sense making. Interacting with toys in whatever way they independently can and exploring their environment by touch, sound, mouth, or vision should be combined with learning through the process of engaging with others to support their efforts to pursue desired outcomes or complete tasks. These are just as important and meaningful for children with severe disabilities as they are for any child.

To date, there have been few studies that have analyzed interventions that focus on sensory experiences for children with severe disabilities. One study investigated children with a diagnosis of cerebral palsy who were able to walk and use speech to communicate (Jameel et al., 2019). The intervention focused on kinesthetic training that helped to significantly improve the participants’ perceptual abilities. Specifically, the invention targeted kinesthetic sensitivity, which is needed to appropriately judge the amount of force needed to lift items, maneuver through the environment, and position one’s body to be successful in everyday activities. Jameel and colleagues used body awareness activities with the children and found that after 36, 30-min sessions the children showed significant improvement in their tactile sense, pressure sense, and cognitive ability. Identifying this connection between sensory input and cognition is an important step towards understanding the lasting impacts of sensation, especially for children with severe disabilities.

In addition to impacting cognition, there is reason to believe that at least some sensory experiences provide opportunities to promote mental health. Sheehy and Nind (2005) discussed the limited literature regarding the mental health and emotional well-being of people with profound and multiple disabilities. They assert that the lack of attention to the mental health of people with multiple disabilities overlooks “their very humanness and their right to quality of life” (2005, p. 35). The authors point to the absence of symbolic communication as a primary reason that the sensory experience and mental health needs of people with multiple disabilities is overlooked, as the lack of conventional communication skills leaves them unheard and misunderstood.

Overall, it is evident that more research is needed to understand the impact of sensory experiences on children with severe disabilities. In the current study, sensory experiences emerged as an important theme during grounded theory thematic analysis that was conducted as part of a larger effort to understand thinking and learning among older children and young adolescents with severe disabilities.

## Materials and Methods

The current study was situated within a three-year postcritical ethnography designed to construct a theory of thinking and learning in students with severe disabilities including complex communication needs (CCN; Erickson et al., 2021). It was approved by the institutional review board at the university where the authors are employed and the school system where the research was conducted. Further, individual adult participants and the parents of the student participants provided written consent. The central question addressed was, *what was the nature and impact of the sensory experiences students with severe disabilities including CCN encountered in their classrooms?*

Postcritical ethnography requires researchers to intentionally reflect on untested assumptions (e.g., that students with severe disabilities must be educated in separate settings) and personal beliefs relative to the study at hand (Noblit et al., 2004). The interdisciplinary team of six researchers who conducted this study had backgrounds in literacy education, special education, early childhood education, augmentative and alternative communication, assistive technology, severe disabilities, occupational therapy, occupational science, and educational policy. The relevant, collective assumptions and beliefs of the research team include views of:

- disability as dis/ability, which challenges the view of disability as a binary concept and recognizes that disability is, in part, socially-constructed (Goodley, 2014);
- education as a path toward a more equitable world; and
- themselves as researchers who are learners-about-students.

### Site and Participants

The school where we conducted this study is located in the southeastern United States and is representative of the separate educational placements of nine in 10 students with severe multiple disabilities across the country (Kleinert et al., 2015; Erickson and Geist, 2016). The school serves more than 50 school-aged students with a range of severe disabilities. The students are taught in multi-grade classrooms of six to eight students. Each classroom is led by a special education teacher who has the support of a full-time teaching assistant. Additional teaching assistants and nurses address students’ personal care needs across multiple classrooms, and full-time speech-language pathologists, physical therapists, and occupational therapists work with the children and teachers. Other teachers (e.g., art, adapted physical education, media) and specialists (e.g., a teacher of children who are blind and visually impaired and a teacher of children who are deaf and hard of hearing) serve students in this school and others in the school system.

The data in this manuscript focus on two middle-school classes that participated in the larger postcritical ethnography across two school years. We selected these two classrooms because they offered groups of students of similar ages and abilities and teaching staff with similar backgrounds and experience. None of the students reported in this manuscript have known hearing or vision loss, but both are known to be underreported among students with complex needs (e.g., Erickson and Quick, 2016). We have intentionally chosen not to highlight or specifically name the individual teachers and teaching assistants. Instead, we forefront the experiences of the students and the systems that impact those experiences. Our goal is to emphasize the role of these systems rather than individual teachers. Throughout, we use pseudonyms for the students in order to emphasize their personhood rather than their diagnosis or perceived deficits.

#### Classroom 1

There were four or five consented students in Classroom 1 depending on the year of the study. All of the students had severe disabilities and used a range of idiosyncratic gestures, vocalizations, and behaviors to communicate. All of the students had CCN and were learning to use graphic symbols and voice output communication devices to communicate with others. The student featured in the data excerpts in this study is Jamie, who was 10 years old at the start of the study. Jamie, age 10 at the start of the study, was a Latino, male student who received special education services under the IDEA eligibility category, Multiple Disabilities. He was almost always in a wheelchair that he could maneuver himself, but teachers often pushed his wheelchair in the classrooms and when moving from one location in the school to another. He vocalized, sometimes touched graphic symbols from the 36 words from the Universal Core vocabulary to communicate, and sometimes reached out or used his eye gaze to communicate. Mostly he used facial expressions to express his joy, boredom, and outright disdain. Jamie loved music and would wave his arms, dancing, circling around in his wheelchair, laughing, grinning, and raising his eyebrows in response to music. By the second year of the study, Jamie was encouraged to be out of his wheelchair for periods of time, which allowed him to crawl on all fours to get to places he wanted to go.

Tom, just shy of 11 years old when we began the study, was a White, male student eligible for special education services under the IDEA category, Multiple Disabilities. He used a wheelchair for mobility, but he was unable to maneuver it himself. At the beginning of the study, he was working on establishing joint attention and participation. Over time, he began using graphic symbols on a laminated sheet. Then, he moved on to a communication notebook that offered about 25 or 30 pages filled with graphic symbols organized by category (e.g., activities, people, and places) that he accessed by pointing to a symbol representing one of the categories on the menu page. A partner then turned to the corresponding page and Tom selected. By the second year of the study, he was also using a voice output communication device that gave him access to 30 items that were represented by graphic symbols. These included words from the Universal Core vocabulary (e.g., WANT, LIKE, NOT, GO, MAKE; Erickson et al., 2021), the names of the teachers in his classroom, and a symbol representing COMMUNICATION NOTEBOOK that he used to request access to the book. Throughout the results, words produced by selecting these graphic symbols are written in all capital letters.

Sophie, age 16 at the start of the study, was a White female student who received special education services under the IDEA eligibility category, Intellectual Disability-Severe. She was alternatively in a stander or a chair with a lap belt, where she often rocked back and forth. Sophie almost always had a red switch in front of her that said, “Yes, that’s the one I want!” when pressed. She also commonly wore noise canceling headphones. Sophie could often be seen with her chin pulled toward her chest and with a furrowed brow. She often lifted one hand and used her long fingers to fiddle with her ear or her eye or her mouth. Sophie was always happy when music was playing.

#### Classroom 2

There were four or five consented students in Classroom 2 at various points in the study. All of the students had severe disabilities and all communicated using a variety of idiosyncratic gestures, vocalizations, and behaviors. All had CCN and access to some form of voice output communication device with graphic symbols to support their communication and occasionally selected one or two words at a time to communicate with others. The two students featured in data excerpts in this study were Cameron and Devan. Cameron, age 11 at the start of the study, was a White male who was eligible for special education in the category, Intellectual Disability-Severe. He had significant seizures, which impacted his attention and often left him fatigued. When he was not fatigued, he was vocal and worked actively to interact with peers in his vicinity. Marcus could walk with the support of an adult, used a therapeutic stroller to travel long distances, and sat in a therapeutic chair with a tray during instruction. Marcus primarily communicated using vocalizations, gestures, and facial expressions. He was learning to use a voice output communication device that displayed 32 words from the Universal Core vocabulary and a variety of cards and printed displays with graphic symbols representing words related to the topic of the lesson.

Devan, aged 10 at the start of the study, was a White, male student eligible for special education services under the IDEA category, Intellectual Disability-Severe. He used a wheelchair for mobility, but he was unable to maneuver the chair himself. He could walk with physical support from an adult and could move around on the floor through a combination of rolling and combat crawling. He had a voice output communication device with 32 graphic symbols representing words from the Universal Core vocabulary. He accessed it by touching the symbols. However, Devan communicated primarily through facial expressions, vocalizations, reaching, and other movements. Devan typically tore, crumpled, and dropped materials within his reach. A social person, he was often smiling broadly, reaching out, or moving toward classmates and others who entered into his immediate environment.

It is important to note that the teachers in this school were highly trained, and the school was well-regarded. The teachers were passionate, enthusiastic, and caring. They came to school each day eager to be with their students. Nonetheless, as detailed in the results, they sometimes failed to engage all of their students, especially when it came to offering rich sensory experiences that met the students’ sensory processing needs.

### Data Collection Methods

The primary means of data collection for the study was participant observation. In addition, informal interview-style interactions occurred with teachers and other school staff seeking clarification and input regarding things that were observed and expectations regarding upcoming classroom and school activities. The content of these interactions was recorded in fieldnotes collected during the participant observations and were reflected upon in research memos. We were unable to interview the students because they did not have the symbolic communication skills required to participate in interviews or to otherwise provide first-person accounts of their perceptions or experiences in ways that we could record.

Fieldnotes were collected during classroom visits conducted from January 2018 to March 2020. Individual members of the research team visited the classrooms approximately once every 2 weeks. Members took detailed notes while observing, then clarified and added detail and commentary to the notes promptly after each observation. In addition, each researcher kept a personal researcher journal containing timely reflections that were shared and discussed in a weekly research team meeting. During these meetings, the team engaged reflexively in questioning their own and one another’s representation of the data.

Observations and interviews were supplemented with artifacts gathered by members of the research team. These included work samples, instructional materials and products, and photographs of the classrooms. Documents such as student Individual Education Programs and school system policies regarding the use of prescribed curricula and assessments also contributed to the body of data informing this study.

### Analysis Methods

Data in this study were analyzed using grounded theory methodology (Charmaz, 2006). This involved coding the data to distill, sort, and compare segments. Throughout this initial coding, memos were written whenever the first author felt it necessary to flesh out data points or thoughts and connections the data brought up. The memos varied in length and were shared with other team members during weekly meetings to get their perspective on emerging ideas and to develop emerging theory.

Once initial coding was done, focused coding began anew as the entire set of fieldnotes were analyzed to identify themes. In this stage, the goal was to start to group the initial coding together into more general themes (Charmaz, 2006). This focused coding then led to thematic coding, resulting in three major themes: (a) students are afforded limited rich sensory experiences, (b) everyday routines make students passive recipients to school, and (c) instructional approaches result in little interaction with extended periods of waiting.

As recommended by Charmaz (2006), all coding and thematic analysis was completed before the literature review in order to minimize the influence of the existing data around this population. As well as delaying the literature review, the first author worked to keep preconceptions that might influence the process in the forefront while tracking the way that they were influencing what was attended to and how it was understood. The authors acknowledge the fact that they approached this work from a western, White, middle or working class, and able-bodied standpoint. The first author is a pediatric occupational therapist, and the second and third authors are educators. All have previous experience working with children with severe disabilities.

## Results

Across the two classrooms, the students with severe disabilities who were the focus of the analysis exhibited a low registration sensory processing pattern (Dunn, 1997, 2007). This fact is relevant to each of the themes. What is unknown is whether these students were born with that pattern or if that pattern was a product of their abilities, environment, and experience. Due to their severe disabilities, these students had limited means of participating in or seeking out sensory experiences within the classroom context. Similarly, they had limited ability to evoke strategies to self-regulate and seek more or less sensory input. The restricted and highly controlled sensory experiences within the classroom contexts kept the students from meeting their neurological threshold, which could have helped them achieve the optimal zone for learning. As described in the following section, low registration sensory processing patterns, the instructional practices, and the environment resulted in long periods of waiting, which served to reinforce the low registration sensory processing patterns.

### Students Were Afforded Limited Rich Sensory Experiences

Across the classrooms, the students were typically physically spread apart from one another in their wheelchairs or standers with few opportunities for independent exploration or independent work. The teachers moved from one student to the next, interacting briefly and moving on. The only purposeful, regularly occurring sensory experience for the students was music. Music was used to mark transitions, fill transition times, and facilitate lessons. Whenever music was used, there was a clear positive effect on the students. For example, when one teacher turned on the music, the result was:

Jamie is in his chair… dancing by himself, smiling, looking upward, shaking his hands. He seems to be enjoying the music. He has a sublime smile. The teaching assistant comes back to dance with him again, and he has a look of utter JOY. He is smiling, laughing, and full of life in a way that I have not seen through the last 30+ minutes. He turns around in his chair to look at the teaching assistant who is moving his chair to dance with him.

Across observations, music was the one activity that resulted in this type of positive reaction from the students. Each one was observed to dance with whatever independent movement they had including arm waving, finger wagging, and tapping of their toes. They also had the highest levels of interaction with teachers when dancing to the music, and they were often observed requesting more music in various unconventional ways, such as vocalizations, eye contact and smiles. Unfortunately, the teachers controlled when the music was on or off, rather than the students. This was likely a result of the fact that music was used to fill time between activities or mark the introduction to a lesson.

Music wasn’t itself viewed as a teaching tool or important sensory experience. Further, when music appeared to be used as an intentional part of a lesson, the connection was not always clear. For example, in one instance a teacher was teaching a lesson focused on the letter, W. While Whitney Houston’s song “I Wanna Dance with Somebody” played in the background, the teacher moved around the room singing, dancing, and holding up a big piece of paper with the letter W written on it and a card with the word WANT and a graphic symbol representing the card. Though the teacher presented the W and word card close to the faces of each student, there was no clear expectation that the students would respond or interact with either the sign or the symbol. Some students reached out to touch them when they were presented, but other than responding to the physical act of touching, no meaning was assigned to the action or the song. Some students seemed to enjoy this lesson, but the goal of the lesson and connection to the music was unclear. The auditory input (i.e., music) along with the gross motor movement (i.e., dancing), appeared to hit the high neurological threshold of sensory input needed to ‘wake’ these students up and get them into the optimal zone for learning, but it was not clear what they were supposed to be learning beyond looking at or touching the printed W and the symbol representing WANT.

When potential opportunities for other forms of sensory experience were noted, they were typically adult-directed. For example, teacher 2 planned for the students to make Valentine’s cards for their family members. The teacher gathered materials (e.g., stickers, glitter, glue) that typically offer students opportunity for sensory exploration with different textures, shapes, and colors while making cards. However, the students did not explore or interact with the materials. Instead, the teachers directed students step by step through making binary choices about materials and their placement on the card. The following exchange offers an example:

The teacher presents a running string of questions in a rhetorical way, “Do you want glitter glue? Or googly eyes? Do you want colors? Help me put glue on the eyes--oh no, they are sticky back. Should we put a smirk down here? Do you want beads on your card? Do you want to put, ‘I’m watching you?’ If you don’t answer, I will start putting stuff on. I’m putting ‘I’ and dotting ‘t’s.”

Cameron responds with smiles, reaches, and shakes his head ‘no’.

The teacher states, “I think you should write, ‘I love you and you better believe it’”. The teaching assistant states, “Your mom will like that.”

Cameron smiles.

The teacher asks, “What about ‘You are the sparkle in my heart’.”

Cameron responded, “Eh”.

The teacher concludes the lesson by saying,” Ok, let’s write that and then let it dry. We need to get ready for lunch”.

Later the card was put in Cameron’s backpack for him to bring it home for his mother. Throughout, Cameron’s access to sensory experience was limited in ways that reflected the adult directed interactions and activities that dominated in both classrooms.

#### Students Were Afforded Limited Gross Motor Activity

Other missed opportunities for sensory experience resulted from limited gross motor movement in the classroom. All but one student in the two classrooms used a wheelchair for mobility, but only one was able to independently maneuver his manual wheelchair and one other had a motorized wheelchair. The remaining students were dependent on others to move them from one location to another when they were in their wheelchairs. Furthermore, the brakes on the manual wheelchairs were often on the back of the chair, presumably to maximize student safety, but eliminating any potential for the students to independently unlock their wheels to allow them to try to move around the classroom. A few students could independently move themselves on the floor by crawling and rolling, but perhaps because they were older children and adolescents, there were few observed opportunities for them to be on the floor. In fact, there was only one recorded instance where a student was noted to independently crawl across the room, with encouragement from the teacher. In this instance, the teacher provided Jamie with extended time and encouraged him to move to his wheelchair after he was taken out of the standing frame. Jamie moved across the room, and pulled himself up and into his wheelchair with minimal assistance. He was then observed wheeling himself back to his spot at the worktable for some free play. The researcher noted that this was the first time in more than a year of observations that Jamie was seen independently moving himself in or out of his wheelchair. As a rule, teachers moved students, transitioning them from one position to another, pushing their wheelchairs to the desired spots, and locking the brakes to when the chairs were in the positions the teachers selected.

Outside of the classroom, more gross motor movement was observed. During one instance, the researcher accompanied students to their adaptive PE class. The clear change in mood observed in the students was repeatedly noted. During the session, each student was given a chance to take a football down to a basket, drop it in, and ring a bell. The students each worked one-on-one with an adult. A researcher worked with Sophie and “she seems to come alive [during PE class] …with only a little encouragement [she] walks the length of the field several times.” The researcher noted another student, who had been whining and crying as a means of complaining all morning, joined in on the fun. Although most students needed full support from teachers, they seemed to have no complaints about the effort it took to walk the length of the gymnasium and ring the bell. This gross motor movement seen in their adaptive PE class, as with music activities, appeared to meet the students’ high neurological threshold, which then helped them engage and participate more actively.

#### Students Were Afforded Limited Touch Experiences

Touch was another sense that was rarely observed in the classroom. Touch was observed during care activities such as feeding, wiping a nose, or when a student needed to be changed or moved from one piece of equipment or another. Touch during those times served a specific purpose, rather than promoting connection. Importantly, students were sometimes observed trying to connect during these goal-directed interactions with teachers. Unfortunately, their efforts were not understood or acknowledged. For example, in one instance, a teaching assistant grabbed a tissue and said to Tom, “Let me wipe your nose.” In reply Tom used his communication device to say, “NO.” When Tom appeared to note that the teaching assistant still had the tissue and looked ready, he added “GO, FINISHED.” The teaching assistant repeated what Tom said but did not appear to make the connection that maybe he was saying he did not want his nose wiped; she then wiped his nose.

Touch is an important means of establishing connections and communicating with others. The students certainly seemed to understand this. At times, students were observed to reach out for other students or the teacher. Sometimes it was clear that the students were pinching or hitting others because they were frustrated, but at other times, they were using touch to connect in a positive way. For example, students reached out to hold hands with each other, and reached to pull themselves closer or gain attention from peers and teachers. Unfortunately, when teachers noticed this touching, they typically interrupted and redirected the students. Often, unlocking the brakes on their chairs and moving them further away. At other times, students were simply told to stop without explanation. This negative response to student sensory seeking patterns was noted to increase student frustration. It also served to reinforce a low registration sensory processing.

### Everyday Routines Made Students Passive Recipients to School

Student passivity throughout the school day may have been a reflection of a general state of low registration sensory processing; however, there was recurring evidence that the students may have learned to be passive as a result of their everyday school experience. Students had very little control over what they did at school. The teachers posed questions, but rarely provided students with the time or means of responding to the questions. When they were offered a means of responding, the answer options were either highly restricted (as an array of 2 or 3 items) or they did not match the content of the question. For example,

The teacher is scurrying around the room but stops long enough to look at Jamie’s face. She says, “You are NOT happy, are you?” She holds up Jamie’s communication board and points to LIKE NOT WANT GO as she says, “Do you LIKE it or NOT like it?” Jamie reaches with his right hand toward NOT, but she is distracted by one of the other students, puts down the communication board, and walks away before Jamie has a chance to reply. Jamie does not appear to be dejected and does not seem to react in any way to not getting his message delivered.

Teachers also talked to students when the students had no means of responding or initiating a different topic. For example, in one observation, a teacher displayed two cards close to Jamie’s face, each presenting a word and graphic symbol. One card had the word and symbol representing GOOD and the other had the word and symbol representing NOT. In the interaction, the teacher said, “They said it was NOT GOOD,” as she moved the cards for NOT and GOOD, respectively. Then she added, “They did NOT like it,” as she moved NOT and then added, “It was NOT GOOD” as she moved the cards for NOT and GOOD again. Throughout this interaction, the teacher controlled the symbols and was just showing him each card and repeating lines that included the two words. Throughout the interaction, Jamie sat with eyes averted while the teacher talked to him.

When students did look in the expected direction or otherwise actively try to engage with the cards and symbols during these interactions, the teachers often reinforced the act of looking or touching rather than the potential meaning of the communication act. This focus on a behavioral response rather than building a communicative interaction may have systematically taught students to be passive recipients across the school day. Other evidence that teachers were not expecting active communication or participation was found in the words and symbols teachers selected to display during these everyday instructional routines. For example, during one language arts activity, the teacher selected cards with the words and graphic symbols representing WHEN and IT. The teacher held the cards up to match her words when she asked, “WHEN did IT start?” There was a clear question, but no way for the student to utilize the symbol cards to respond given the choice of WHEN and IT. On another occasion, a teacher selected the cards with the words and symbols representing WHAT and WEATHER. As she held up the cards she asked, “WHAT is WEATHER?” Sophie reached for the card representing WHAT. The teacher did not acknowledge her reach or the fact that Sophie said, “Ma ma ma.” Instead, the teacher pulled out a single message voice output device programmed to say, “Yes, that’s it.” She put the device in front of Sophie who quickly responded by touching it. The teacher repeated, “Yes, that’s it!” and moved on to the next student. Sophie responded as expected, but the response did not generate any meaning or ongoing interaction that may have helped her shift from a passive to an active role.

There were times when teachers asked yes/no questions or offered choices and worked with students to try to find a means of responding that led to mutual understanding and ongoing interaction. For example, during one observation, the teacher was trying to get Devan to make a choice between two books.

The teacher holds up one book at a time in front of Devan and says, “Devan, do you want to read the ABC book? Use this arm (touching one of Devan’s arms). Use your words. Do you want to *read The Cat in the Hat*?” Devan laughs and reaches out to the book. The teacher responds by continuing to hold up one book at a time saying, “Do you want *One Fish Two Fish Red Fish Blue Fish*?” Devan laughs and reaches out again … “or do you want *Green Eggs and Ham*?” The teacher taps Devan’s arm with book, Devan reaches out to touch the book. The teacher still does not recognize the reaching behavior and says, “One more time, Devan.” Then the teacher holds up the first book again saying, “ABC book?” Devan responds by laughing and putting his head down.

Throughout the day, students demonstrated a low registration sensory processing pattern. When they did respond to their teacher’s direct requests or bids for attention, their efforts rarely resulted in ongoing interactions or active participation. Overall, there were few instances when the everyday routines encouraged or supported more active engagement or patterns of sensory processing.

### Instructional Approaches Resulted in Little Interaction With Extended Periods of Waiting

The way teachers organized and controlled the classrooms resulted in students spending a great deal of time sitting and waiting throughout the day. They waited to be moved, to be touched, to be interacted with, to be talked to, and to be given something to do or attend to. Often, they waited while their peers had a turn. As one researcher observed, “the other kids have to sit and wait the whole time the teacher is cycling through with the other kids. It would be so easy [for the students] to check out.” It appeared that these students did “check out” of the lesson, as the researcher noted, but they were regularly observed looking around the room at the teachers coming and going, chewing on their fingers, reaching out to touch a peer, rocking back and forth and more. The limited structures of interaction observed in the classroom lead the students to find other ways to engage themselves.

During one lesson, Cameron continually looked around the room and fidgeted. It appeared that he was unengaged in the lesson. He communicated his apparent boredom in a few ways, as illustrated by the following excerpt from the fieldnotes. His teacher was reading a book aloud to the class, and Cameron was seated in a therapeutic chair with a lap belt and an empty desk in front of him.

Cameron looks up and watches as the teacher is reading. He looks to the right toward the teaching assistant, or maybe he is just looking down. He waves his left arm left toward another researcher and bangs on the table three times. Cameron looks left toward me and checks me out, watching me type. He waves his head side to side in a ‘no’ motion, then rubs his left arm on his tray. He puts his finger in his mouth then looks over at me. He looks up to the left…Cameron continues looking to his left and putting his hand in his mouth.

Many of Cameron’s behaviors could be characterized as *stimming*, which is a self-stimulatory behavior that is marked by a repetitive action or movement of the body (Stimming, n.d.). However, the question here is whether he was engaging in “stimming” or was he just trying to fulfill his sensory needs given limited options. This type of behavior could easily be classified as sensory-seeking. Across multiple observations, the students were reported to rock back and forth, bite their fingers, look at the teachers moving around the room and in the hallway, and engage in other behaviors that could collectively be classified as sensory-seeking. In each of these instances, a lesson was going on, however, the lessons offered few opportunities for students to engage, interact, or otherwise meet their neurological thresholds. As a result, the students appeared to find other ways to meet them.

## Discussion

Understanding the impact of sensory experiences relative to sensory processing patterns is important. In the case of children with severe disabilities and CCN, sensory experiences and opportunities are especially important given their limited ability to self-regulate and either seek or reduce sensory input in a way that matches their neurological thresholds. As described in the current study, limited sensory experiences might contribute to what *presents* as a low register, passive sensory pattern among many children with severe disabilities and CCN; however, a closer look at what is often classified as stimming behavior may suggest that at least some of these children are seeking higher levels of sensory input to meet their needs.

Severe disabilities can interfere with the development of sensory processing and the ability to enact active patterns in response to sensory input. The student participants in this study had limited mobility, which made it difficult for them to engage actively in sense making. With these students and others with severe disabilities, intentional efforts to support participatory sense making (Di Paolo and De Jaegher, 2012) may offer much-needed sensory experience. Regular observations of the student participants “waking up” when there was music, dancing, and gross motor activity, suggest that the student participants in this study benefited when these efforts were made. Further the students’ responses during these interactions supports the assertion that students who otherwise appeared to have high neurological thresholds for sensory input with passive self-regulation patterns may, in fact, have learned to be passive in the face of repeated, limited sensory experience. During these instances of more intensive sensory input, the student participants socially interacted with the teacher and teaching assistant. They vocalized, laughed, and appeared eager to exert effort and participate. The general mood changes that resulted from vestibular input were repeatedly documented in fieldnotes. For example, Jamie’s affect, participation, and engagement all improved when dancing in his chair and with the teaching assistant. Although this did not change Jamie’s low registration, the gross motor movement, one-on-one attention, and apparent interest in the music aroused Jamie and other students. Increasing the amount of movement and vestibular input children with severe disabilities receive throughout the day may lead to a shift in register in the long term.

Other sensory experiences included touch and vestibular input. The teachers would grab the students’ hands, sometimes help them stand up, and sway them back and forth. This was one of the only times that touch was used for purely social interaction. Other than during dancing, touch focused on goal-directed duties required to meet the students’ personal care needs. As in the example of the teaching assistant wiping Tom’s nose, touch was used to address needs as perceived by the teachers, which eliminated students’ bodily autonomy. Tom was a middle school student. It would have been reasonable to provide Tom with a choice regarding who touched him and how, especially since Tom very clearly used a communication device with efficiency and accuracy to express his desire not to be touched. Unfortunately, the teaching assistant either did not understand or respect Tom’s communication efforts, as she simply repeated his words rather than responding to them meaningfully. The teaching assistant may have felt it was important to everyone’s health and hygiene to wipe Tom’s nose, but by not explaining this to him, she was reinforcing Tom’s low registration sensory processing pattern.

Despite years of schooling and at least two years during the current study with restricted sensory experience, the student participants persisted in seeking sensory input, connection, and communication. There were numerous occasions when students attempted to interact with one another, when they danced in their locked wheelchairs, and when they quietly engaged in behaviors that could be interpreted as stimming that provided sensory experience. Unfortunately, these efforts were unnoticed, ignored, or interrupted by the teachers in the classrooms. There were many instances of missed opportunities of communication, interaction, lost bids for attention, and teachers physically directing, or controlling students rather than seeking to understand them. It is important that teachers work to understand all of their students’ communication efforts while helping them develop the symbolic communication skills that Sheehy and Nind (2005) warn are critical to ensuring the mental health of people with severe disabilities.

Active engagement and interaction are central elements of effective symbolic communication development for children with severe disabilities and CCN (Erickson et al., 2021). Unfortunately, one of the most salient themes that appeared in the field notes was the amount of time the students spent sitting and waiting. Each of us spends time each day waiting—waiting for the toaster to pop, the light to change, or the lecture to get started. But in these classrooms, the student participants spent a disproportionate amount of time waiting. This waiting typically ensued without interruption given the students’ apparent low registration processing pattern. Without the students actively seeking input through gross movement or loud vocalizations, there was no impetus for teachers to shorten the periods of waiting. Instead of working to improve student sensory processing and optimize the environment for student learning and engagement, teachers were being reinforced by students’ low registration, which then led teachers to reinforce students’ passive, low registration patterns.

It is unclear if the students’ low registration sensory processing patterns were learned or innate, but the classrooms in this study definitely reinforced this low registration sensory processing pattern over a sensory seeking one. The students could not engage in many sensory seeking behaviors, as they sat in locked wheelchairs far enough away from one another to eliminate opportunities for physical interaction. However, there was evidence that they engaged in self-stimulatory behaviors, stimming, that provided sensory input when the environment did not. This suggests that perhaps they were innately driven to seek sensory input and that the low registration pattern had been learned and reinforced over time. It also points to the need for teachers to provide more opportunities for participatory sense-making.

## Implications

Improving outcomes for students with severe disabilities requires that educational teams attend to their sensory needs. Though more research is needed to understand the impacts of sensory and play based learning in students with severe disabilities, the current study provides important initial evidence of the need to inform teachers of the impact of limited sensory input and different sensory processing patterns. Professional development courses could be a way to help teachers understand the senses and the impact of purposeful sensory experiences on learning, motivation, and self-regulation. This could also be accomplished by occupational therapists who provide direct and indirect services to students. These professionals could help teachers understand and apply the model of sensory processing by Dunn (1997). This would allow educational teams to identify patterns of individual students and offer specific strategies to manage sensory experiences to maximize student engagement and participation throughout the school day. Understanding the different types of sensory processing and how to help each type, especially low registration, get to the optimal zone could also significantly improve the experience, engagement and interactions between teachers and the students.

## Conclusion

The limited rich sensory experiences observed in these two middle school classrooms have a profound impact on all students, but especially students with severe disabilities who may be unable to meet their own sensory input needs due to physical and environmental constraints. Without environments and other people to help them meet their sensory needs, the students are more likely to establish passive sensory processing patterns, which then reinforce increasingly long periods of waiting and more passivity. A low registration sensory processing pattern compounds the impacts of learning environments that offer few sensory experiences, and more research is needed to understand how to increase purposeful sensory experiences and the impact these experiences can have on students with severe disabilities.

## Data Availability Statement

The original contributions presented in the study are included in the article/supplementary material, and further inquiries can be directed to the corresponding author.

## Ethics Statement

The studies involving human participants were reviewed and approved by Office of Human Research Ethics at UNC-CH. Written informed consent to participate in this study was provided by the participants’ legal guardian/next of kin.

## Author Contributions

SA, KE, and CD’A contributed to the conception of the analysis for the current manuscript. KE was the PI and CD’A the project director of the larger project. KE and CD’A collected the data, contributed substantially to the methods, and revised all sections of the manuscript. SA analyzed the data with support from KA and CD’A and wrote the first draft of this manuscript. All authors contributed to the article and approved the submitted version.

## Funding

The research reported in this article was made possible in part by a grant from the Spencer Foundation (#201800037). The views expressed are those of the authors and do not necessarily reflect the views of the Spencer Foundation.

## Conflict of Interest

The authors declare that the research was conducted in the absence of any commercial or financial relationships that could be construed as a potential conflict of interest.

## Publisher’s Note

All claims expressed in this article are solely those of the authors and do not necessarily represent those of their affiliated organizations, or those of the publisher, the editors and the reviewers. Any product that may be evaluated in this article, or claim that may be made by its manufacturer, is not guaranteed or endorsed by the publisher.
